# Simultaneous Cervical and Lumbar Spinal Cord Stimulation Induces Facilitation of Both Spinal and Corticospinal Circuitry in Humans

**DOI:** 10.3389/fnins.2021.615103

**Published:** 2021-04-20

**Authors:** Behdad Parhizi, Trevor S. Barss, Vivian K. Mushahwar

**Affiliations:** ^1^Neuroscience and Mental Health Institute, University of Alberta, Edmonton, AB, Canada; ^2^Sensory Motor Adaptive Rehabilitation Technology (SMART) Network, University of Alberta, Edmonton, AB, Canada; ^3^Division of Physical Medicine and Rehabilitation, Department of Medicine, University of Alberta, Edmonton, AB, Canada

**Keywords:** transcutaneous spinal cord stimulation, corticospinal facilitation, H-Reflex, motor evoked potential (MEP), cervico-lumbar coupling, interlimb coordination, locomotion, spinal cord injury

## Abstract

Coupling between cervical and lumbar spinal networks (cervico-lumbar coupling) is vital during human locomotion. Impaired cervico-lumbar coupling after neural injuries or diseases can be reengaged via simultaneous arm and leg cycling training. Sensorimotor circuitry including cervico-lumbar coupling may further be enhanced by non-invasive modulation of spinal circuity using transcutaneous spinal cord stimulation (tSCS). This project aimed to determine the effect of cervical, lumbar, or combined tSCS on spinal reflex (Hoffmann [H-]) and corticospinal (motor evoked potential [MEP]) excitability during a static or cycling cervico-lumbar coupling task. Fourteen neurologically intact study participants were seated in a recumbent leg cycling system. H-reflex and MEP amplitudes were assessed in the left flexor carpi radialis (FCR) muscle during two tasks (Static and Cycling) and four conditions: (1) No tSCS, (2) tSCS applied to the cervical enlargement (Cervical); (3) tSCS applied to the lumbar enlargement (Lumbar); (4) simultaneous cervical and lumbar tSCS (Combined). While cervical tSCS did not alter FCR H-reflex amplitude relative to No tSCS, lumbar tSCS significantly facilitated H-reflex amplitude by 11.1%, and combined cervical and lumbar tSCS significantly enhanced the facilitation to 19.6%. Neither cervical nor lumbar tSCS altered MEP amplitude alone (+4.9 and 1.8% relative to legs static, No tSCS); however, combined tSCS significantly increased MEP amplitude by 19.7% compared to No tSCS. Leg cycling alone significantly suppressed the FCR H-reflex relative to static, No tSCS by 13.6%, while facilitating MEP amplitude by 18.6%. When combined with leg cycling, tSCS was unable to alter excitability for any condition. This indicates that in neurologically intact individuals where interlimb coordination and corticospinal tract are intact, the effect of leg cycling on cervico-lumbar coupling and corticospinal drive was not impacted significantly with the tSCS intensity used. This study demonstrates, for the first time, that tonic activation of spinal cord networks through multiple sites of tSCS provides a facilitation of both spinal reflex and corticospinal pathways. It remains vital to determine if combined tSCS can influence interlimb coupling after neural injury or disease when cervico-lumbar connectivity is impaired.

## Introduction

The recent surge of investigations in modulating the circuitry of the spinal cord by means of non-invasive transcutaneous spinal cord stimulation (tSCS) suggests that this approach has the potential to facilitate improved sensorimotor rehabilitation (Balykin et al., 2017; Inanici et al., 2018). Applying tSCS at either the cervical or lumbar level of the spinal cord has been shown to enhance upper and lower limb motor function and mitigate spasticity in persons with spinal cord injury (SCI) (Gerasimenko et al., 2015a; Inanici et al., 2018; Sayenko et al., 2019; Hofstoetter et al., 2020). Epidural spinal cord stimulation (eSCS) and tSCS may activate similar neural structures, and computer modeling and evoked electrophysiological responses suggest the likely involvement of primary afferent fibers of the posterior root in evoking motor outputs (Ladenbauer et al., 2010; Danner et al., 2011; Hofstoetter et al., 2018). eSCS improves spinal motor output and volitional movements even in cases of severely reduced supraspinal input (Herman et al., 2002; Carhart et al., 2004; Harkema et al., 2011; Mayr et al., 2016; Wagner et al., 2018). Most recently, eSCS applied to the lumbar spinal cord, in conjunction with intensive locomotor training, allowed persons with clinically complete SCI to walk over ground for short distances (Angeli et al., 2018; Gill et al., 2018). This demonstrated that dormant neurons that survived the injury may be reengaged with spinal neuromodulation and produce stepping-like movements (Courtine et al., 2009; Angeli et al., 2014).

The coordination between the legs and arms is an inherent feature of locomotor neural networks (Zehr et al., 2016) with coupling between cervical (arms) and lumbar (legs) spinal networks (cervico-lumbar coupling) well demonstrated in both animals and humans (Yamaguchi, 1986; Juvin et al., 2005). Oscillatory movements are governed by separate locomotor centers known as central pattern generators (CPG) located in the cervical and lumbar spinal cord segments (Zehr et al., 2016; Frigon, 2017). In mammalian quadrupedal locomotion, coordinated rhythmic movements of the forelimbs and hindlimbs are mediated primarily by inter-CPG connections (Ballion et al., 2001; Juvin et al., 2005; Gordon et al., 2008). In animal models, the hindlimbs can modulate neural networks associated with the forelimbs, and vice versa (Ballion et al., 2001). Similarly, to quadrupedal mammals, a bidirectional linkage between the cervical and lumbar segments of the spinal cord during rhythmic movements is present in humans (Dietz, 2002; Zehr et al., 2009), facilitated primarily by propriospinal connections (Frigon et al., 2004; Ferris et al., 2006).

Coupling between the arms and legs in humans has been demonstrated by the suppression of H-reflexes evoked in one limb by rhythmic movements of the remote limbs (Ferris et al., 2006; Hundza and Zehr, 2009; Zhou et al., 2018b). Moreover, engaging cervico-lumbar connections with simultaneous arm and leg (A&L) cycling training has been shown to improve walking after both chronic incomplete SCI (Zhou et al., 2018a) and stroke (Klarner et al., 2016a,b). Strikingly, the addition of the arms in A&L cycling training appears to transcend gait-specific training strategies including treadmill and over-ground locomotor training by doubling the magnitude of improvements in walking parameters (Zhou et al., 2018a). Highlighting the importance of these interlimb connections, arms-only cycling has also been shown to improve over ground walking function after stroke (Kaupp et al., 2018). The substantially larger functional improvements experienced after A&L training relative to gait-specific training are therefore at least partially rooted in the reengagement of cervico-lumbar connections.

Corticospinal projections to spinal motor neurons are generally facilitated during cycling in neurologically intact individuals. However, this facilitation was not present during arm cycling for individuals with incomplete SCI prior to A&L cycling training (Zhou et al., 2017). Excitingly, 12 weeks of A&L cycling training reengaged these connections by significantly increasing the amplitude of the motor evoked potential (MEP) in the tibialis anterior muscle compared to baseline levels prior to the intervention (Zhou et al., 2017). Furthermore, disruptions in cervico-lumbar connectivity, which are noted after both incomplete SCI and chronic stroke, can be reduced by the simultaneous A&L cycling paradigm (Klarner et al., 2016b; Zhou et al., 2018b). Presynaptic inhibition of Ia afferent terminals (pre-motorneuronal level) is thought to exert such an effect (Gossard, 1996; Frigon et al., 2004; Nakajima et al., 2013). Therefore, the coupling of cervical and lumbar networks, mediated by both ascending and descending propriospinal connections, is vital to interlimb coordination and the restoration of walking after neural injury (Laliberte et al., 2019).

Enhancing cervico-lumbar connectivity by pairing A&L cycling with tSCS may further improve mobility outcomes (Pradarelli et al., 2020). Recently, we showed that cervical tSCS significantly suppresses the soleus H-reflex (the remote limb) (Barss et al., 2019), similarly to the effect of arm cycling on the soleus H-reflex. Benavides and colleagues showed that after 20 min of tSCS, the amplitude of subcortical MEP (i.e., cervicomedullary evoked potentials or CMEP) increased, but not the amplitude of MEPs (Benavides et al., 2020). They determined that tSCS causes an increase in intracortical inhibition (measured by paired stimuli conditioning) that restricted the cortical MEPs. The results imply that the effect of tSCS varies between cortical and spinal networks, inhibiting the former and facilitating the latter. Moreover, ulnar nerve stimulation has been shown to potentiate spinally evoked motor responses (M-wave) (evoked by single pulse tSCS at a level between the lower thoracic and upper lumbar) across multiple muscles of the lower limb in both neurologically intact and spinal cord injured individuals (both complete and incomplete) (Atkinson et al., 2020). This signifies that conditioning of descending interlimb projections to lumbosacral motor pools occurs at least in part by similar networks that are activated with tSCS. Moreover, paired tSCS at the L2 and S1 segments of the spinal cord resulted in potentiation of the evoked response from either site alone, indicating synergistic effects of multi-segmental pathways (Sayenko et al., 2015). Preliminary reports in abstract form have shown that combined cervical and lumbar tSCS may improve locomotor function, sensation, and bladder function in a single participant when combined with intensive physical therapy (Pradarelli et al., 2020). However, little information is known about how cervical or lumbar tSCS influences the excitability of corticospinal and spinal networks in the upper limb, and how it alters interlimb coupling. It also remains unknown if synergistic effects of multi-segmental tSCS occur between the cervical and lumbar segments of the spinal cord. This lack of information limits the translational ability of tSCS and highlights several key issues that need to be addressed prior to the appropriate implementation of tSCS into rehabilitation strategies.

The primary purpose of this study was to determine the extent to which cervical spinal and corticospinal circuitry can be modulated by either cervical or lumbar tSCS during a static task. Secondarily, this study aimed to establish whether combined cervical and lumbar tSCS further facilitates neuromodulation of spinal and corticospinal circuitry compared to either site alone. Finally, this study aimed to determine whether tSCS influences cervico-lumbar connectivity and corticospinal excitability during a rhythmic task (leg cycling). Based on previous work, we hypothesized that tonic activation of the lumbar spinal cord through tSCS would significantly inhibit the activity of cervical networks but increase the excitability of the corticospinal tract. We also hypothesized that simultaneous stimulation of cervical and lumbar networks would further enhance the effects seen with either site alone. Addressing these hypotheses will shed light on how the neural control of interlimb coordination may be most effectively facilitated by tSCS.

## Materials and Methods

### Experimental Design

**FIGURE 1:**
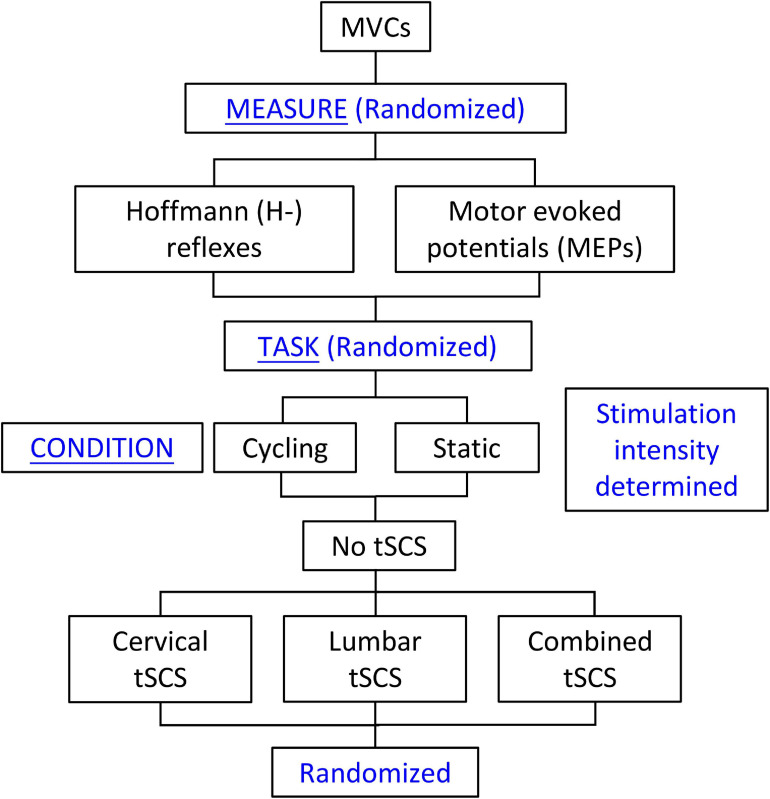
Study design. Maximal voluntary contractions were collected and amplitudes of cervical and lumbar tSCS were determined at the beginning of each experiment. This was followed by random selection of the Hoffmann (H-) reflex or motor evoked potential (MEP) measures. The cycling or static tasks were then randomly selected. For each of these tasks, the No-tSCS condition was tested first, followed by random selection of the remaining conditions (Cervical tSCS, Lumbar tSCS, and Combined tSCS). The No tSCS condition was completed first in order to obtain the stimulation amplitudes to the median nerve and TMS needed to evoke the H-reflex and MEP, respectively, to be used across all conditions.

Building on our previous work (Barss et al., 2019), this project aimed to determine the effect of cervical, lumbar, or combined tSCS on spinal (H-reflex) and corticospinal (MEP) excitability during a static or cycling task. The neuromodulatory effects of tSCS were assessed in neurologically intact study participants who were seated in a recumbent leg cycling system. H-reflexes and MEPs were assessed in the FCR muscle of the left arm during two tasks (static and leg cycling) and four conditions: (1) No tSCS, (2) tSCS applied to the cervical enlargement (Cervical); (3) tSCS applied to the lumbar enlargement (Lumbar); and (4) simultaneous cervical and lumbar tSCS (Combined) (Figure 1). Thus, separate trials assessed H-reflex and MEP excitability during eight conditions for each study participant (16 conditions total).

### Experimental Setup

**FIGURE 2:**
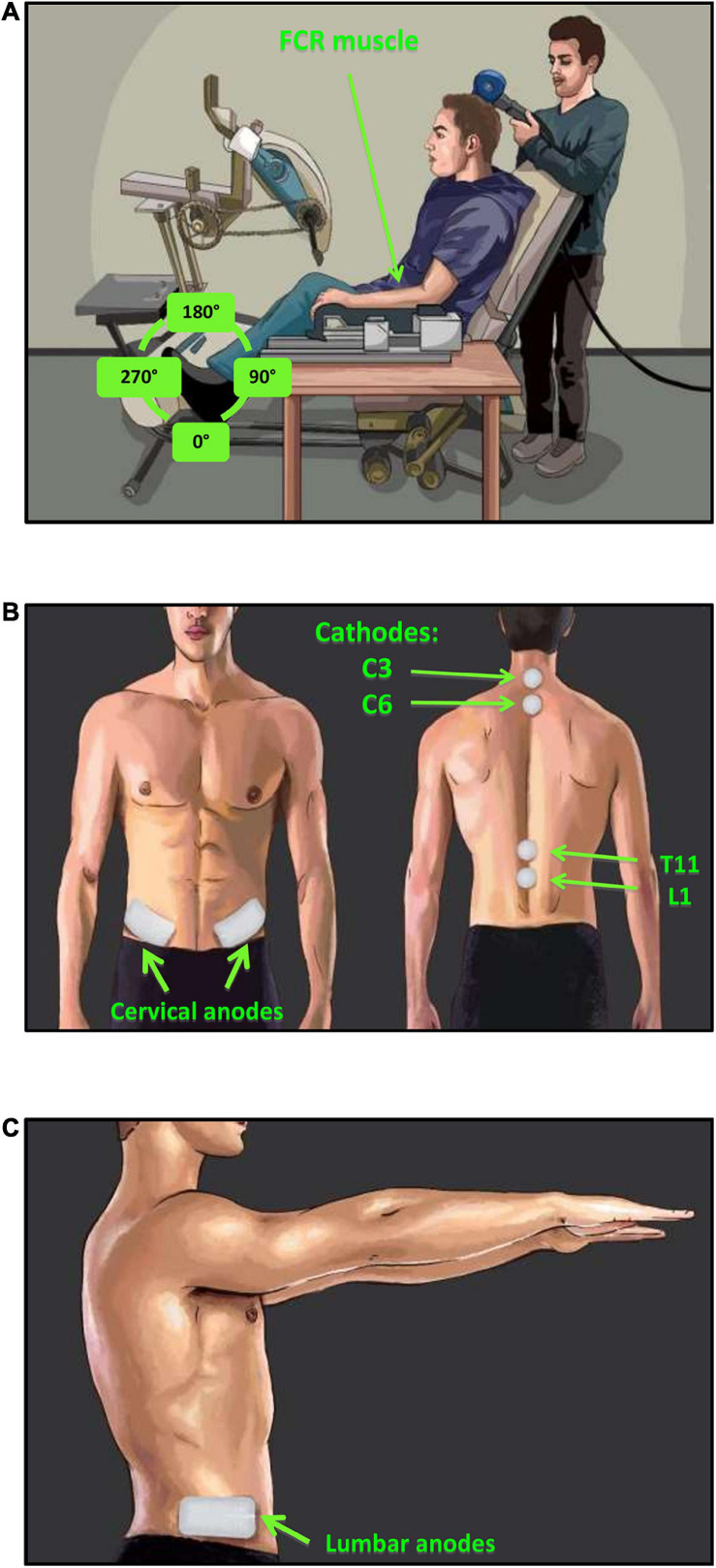
Experimental setup and tSCS electrode placement. **(A)** H-reflexes were evoked via stimulation of the median nerve and recorded in the FCR muscle during a consistent baseline contraction of 5–10% maximum voluntary force (MVF). MEPs were evoked via TMS of the motor cortex and recorded in the FCR muscle during consistent baseline contraction of 5–10% MVF. **(B)** tSCS was delivered via two 2.5 cm round cathodic electrodes placed midline at C3–4 and C6–7 (cervical) or T11 and L1 (lumbar) spinous processes. Two rectangular anodic electrodes were placed bilaterally over the iliac crests, **(C)** in addition, two extra anode electrodes were place laterally beside the first two to accommodate simultaneous cervical and lumbar tSCS condition.

Participants were seated in a custom-adapted leg cycling ergometer (ERGYS 2, Therapeutic Alliances, Fairborn OH) with a fixed back support and movable seat to accommodate for participants’ height (Figure 2A). The left and right side of each leg crank were linked with 180-degree phase difference. The torso was restrained using a seat belt, and the experimental arm (left) was secured in a fixed pronated position using straps into a secure brace, embedded with a force sensor (Neurolog, Hertfordshire, United Kingdom). The left arm was chosen to be comparable to previous investigations from the same laboratory (Zhou et al., 2017, 2018b). The effect of leg cycling on upper limb reflex responses between the right and left limbs, and the relative difference between experimental conditions is expected to be similar regardless of which arm is used (Zehr et al., 2007; Nakajima et al., 2013). Participants were instructed to maintain the same position throughout the experiment and to place the non-experimental arm on the right armrest of the chair. The 180° position of the left leg (i.e., 12 o’clock) was chosen as the phase of the leg during which both H-reflexes and MEPs are evoked for both the leg static and cycling conditions. This placement of the leg was chosen based on previous studies indicating that the 180° leg position produces both peak muscle activity and the largest inhibition of the FCR H-reflex (Zhou et al., 2017, 2018b). Therefore, the positions of the left and right leg were held constant at 180 and 0°, respectively, during static trials (Figure 2A). During the cycling trials, participants performed counter-clockwise rhythmic leg cycling (viewed from the left) loaded with a resistance equivalent to 50% of the ergometer’s maximal resistance at a constant frequency of ≈1 Hz (∼60 rpm) (Zehr, 2002). Online visual feedback of cycling speed was provided on a monitor in front of them.

### Participants

Fourteen (14) neurologically intact participants completed the H-reflex (3 female, 11 male) and MEP (4 female, 10 male) assessments, with 11 completing both protocols. Because 3 individuals were excluded from MEP assessment due to possible contraindications to transcranial magnetic stimulation (TMS), three additional participants were recruited to complete only the MEP portion of the protocol. Participants signed an informed written consent form prior to their participation in the study. The study protocol was approved by the University of Alberta Human Research Ethics Committee. All participants were verbally instructed about the experimental procedures and completed a safety questionnaire about the use of TMS.

### Transcutaneous Spinal Cord Stimulation

**FIGURE 3:**
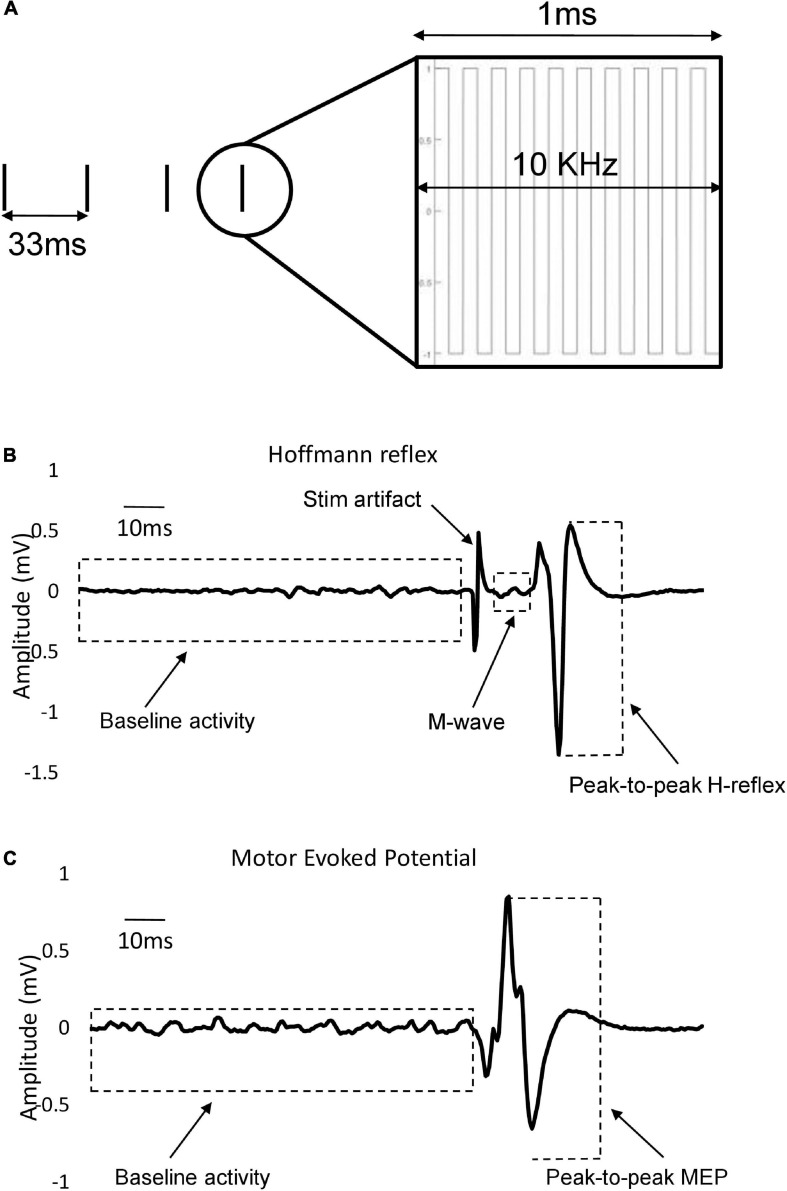
Stimulation parameters and waveforms. **(A)** Stimulation pattern: Envelops of 1 ms-long burst of 10 kHz square-wave biphasic pulses carried at the rate of 30 Hz. **(B)** Representative example of individual evoked FCR H-reflex trace that also encompasses M-wave, stimulation artifact and baseline activity. Different components are measured in a 400-ms window (starting at 100 ms pre-stimulus to 300 ms post-stimulus). Peak-to-peak H-reflex amplitude used in data analysis is shown with dashed lines. **(C)** Single example of recorded FCR MEP trace with pre-stimulus baseline activity. Peak-to-peak MEP amplitude used in data analysis is shown with dashed lines.

Transcutaneous stimulation of the spinal cord was delivered by a constant current stimulator (NEOSTIM-5, Cosyma Ltd., Moscow, Russia) through two adhesive 2.5 cm round cathodic electrodes (Axelgaard Manufacturing Co., Ltd., United States) placed midline at C3–4 and C6–7, and T11 and L1 spinous processes for activating the cervical and lumbar regions of the cord, respectively (Figure 2B). Two 5 cm × 10 cm rectangular electrodes were placed bilaterally over the iliac crests as anodes (Figure 2B) for the cervical tSCS while two additional anode electrodes were placed laterally for the lumbar tSCS (Figure 2C). In total, four anodic electrodes corresponding to four cathodic electrodes were used to ensure that the cervical and lumbar channels were isolated during combined stimulation. The tSCS waveform consisted of 1 ms-long trains of 10 kHz biphasic square pulses repeated at a frequency of 30 Hz (Figure 3A).

To identify maximal intensity, tSCS amplitude was increased in 1 to 5 mA increments to the point when the participants reported their tolerance capacity. At this intensity, participants felt a strong buzzing, fluttering, or vibration sensation at the cathodic site. However, the sensation was free from pain with little to no sensation at anodic sites. There were no evoked M-wave in the arm or leg due to tSCS in the current work. This approach to identifying the maximal tolerable intensity was used to ensure relative similarity in stimulation intensity between individuals compared to the threshold intensity used in our previous investigation (Barss et al., 2019).

The intensity of tSCS across all participants for H-reflex assessment was 50.4 ± 10.7 mA at the cervical level and 41.3 ± 11 mA at the lumbar level across all conditions. The tSCS intensity for MEP assessment was 51.7 ± 10.5 mA at the cervical level and 42.2 ± 11.3 mA at the lumbar level across all conditions. Table 1 provides the tSCS amplitudes for all participants at the cervical and lumbar sites. Stimulation was initiated 30 seconds to 1 min prior to each condition, remained on during the course of each condition and was turned off immediately after the recording of H-reflexes/MEPs was completed. Recordings of H-reflexes and MEPs lasted about 2–3 min. Therefore, including the time prior to data collection, tSCS remained on for 3–4 min during each condition. The stimulation was turned off between conditions and a break of 2–3 min was given to reduce fatigue or summation effects of tSCS.

**TABLE 1:** Summary of tSCS amplitude across study participants.

**Hoffmann reflex (H-reflex)**						
**Amplitude (mA)**	**tSCS amplitude for each participant (*n* = 14)**					
	*Cervical*	P1 = 65	P2 = 42	P3 = 40	P4 = 37	P5 = 50
		P7 = 55	P9 = 70	P10 = 50	P11 = 50	P12 = 55
		P14 = 35	P15 = 44	P17 = 65	P19 = 48	**Mean: 50.43** **SD: 10.72**
	*Lumbar*	P1 = 48	P2 = 40	P3 = 40	P4 = 29	P5 = 32
		P7 = 44	P9 = 55	P10 = 50	P11 = 38	P12 = 48
		P14 = 27	P15 = 30	P17 = 65	P19 = 33	**Mean: 41.36** **SD: 11.01**

**Motor evoked potential (MEP)**						

**Amplitude (mA)**	**tSCS amplitude for each participant (n = 14)**					

	*Cervical*	P1 = 65	P2 = 42	P4 = 37	P5 = 50	P7 = 55
		P9 = 70	P11 = 50	P14 = 35	P15 = 44	P17 = 65
		P19 = 48	P20 = 50	P21 = 60	P22 = 53	**Mean: 51.71** **SD: 10.53**
	*Lumbar*	P1 = 48	P2 = 40	P4 = 29	P5 = 32	P7 = 44
		P9 = 55	P11 = 38	P14 = 27	P15 = 30	P17 = 65
		P19 = 33	P20 = 50	P21 = 52	P22 = 48	**Mean: 42.21** **SD: 11:36**

### Hoffmann (H-) Reflex

The FCR H-reflex was evoked by stimulating the median nerve near the cubital fossa using bipolar electrodes with square wave pulses (1 ms-long). The electrical stimulation was delivered using a constant current stimulator (Digitimer model DS7A, Medtel, NSW, Australia) with 5–8 s of inter-stimulation interval. A minimum of 3 s between each random stimulation is recommended for evoking H-reflexes to avoid post-activation depression (Rossi-Durand et al., 1999; Zehr, 2002). For each trial during the static condition, participants held a consistent low-level contraction of FCR muscle between 5 and 10% of their maximum voluntary force (MVF). MVF was defined as the highest isometric force recorded during maximum voluntary contractions (MVCs). To acquire the MVF, each participant completed three trials of maximal voluntary wrist flexion while the force was measured with a force transducer. MVF was then used as a reference to set a target for background contraction. The equivalent of 5–10% of the measured MVF was displayed on an oscilloscope for visual feedback to maintain the same level of contraction throughout all conditions of the experiment. This was done to ensure similar descending drive to spinal motoneurons throughout all experimental trials, and the choice of target force in this range was based on each individual’s comfort to track and maintain the chosen level of background contraction throughout the experiment. During cycling conditions, a position sensor tracked the left leg rotational angle. Stimulation for H-reflex assessment was delivered at the 180° position (with reference to the left leg) (Figure 2A) every 5–8 revolutions. A total of 10 stimuli were delivered for each experimental condition.

**FIGURE 4:**
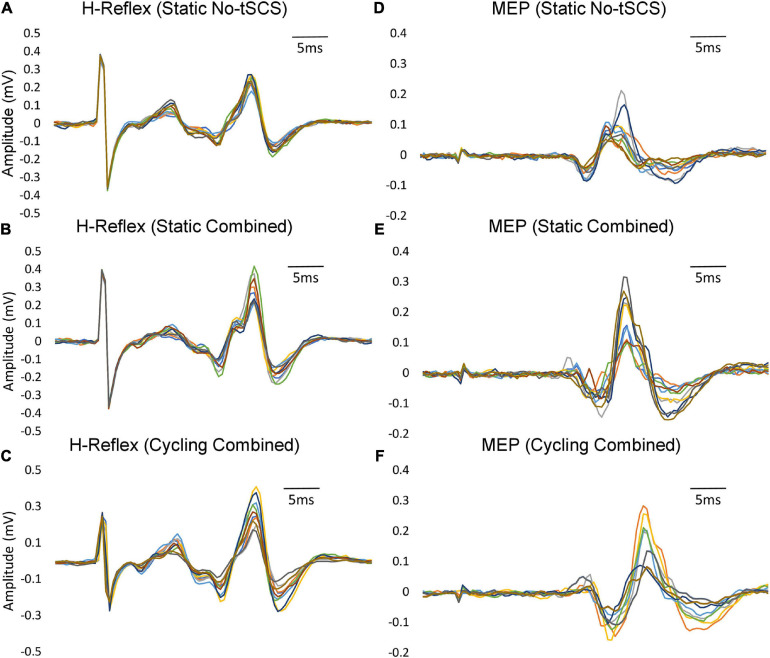
Variability within individual participants. Examples of FCR H-reflex and MEP responses (10 sweeps) from an individual participant within a condition are provided. **(A)** Static H-reflexes evoked with No-tSCS. **(B)** Static MEPs evoked with No-tSCS. **(C)** Static H-reflexes evoked with Combined tSCS. **(D)** Static MEPs evoked with Combined tSCS. **(E)** Cycling H-reflexes evoked with Combined tSCS. **(F)** Cycling MEPs evoked with Combined tSCS.

**FIGURE 5:**
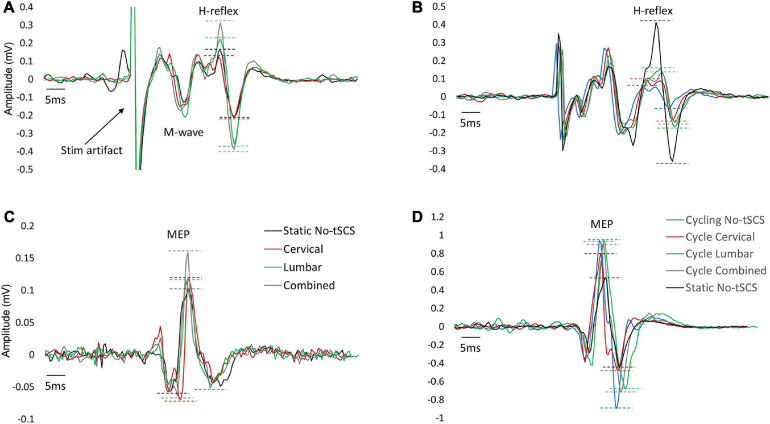
Typical FCR H-reflex and MEP traces across conditions. An example of FCR H-reflex and MEP responses from one participant across all conditions. **(A)** Changes in H-reflex amplitude with Cervical, Lumbar and Combined tSCS compared to No-tSCS while the legs were held static. **(B)** Suppression of H-reflex amplitude across all cycling conditions compared to legs static, No-tSCS. **(C)** Changes in MEP amplitude with Cervical, Lumbar and Combined tSCS compared to No-tSCS while the legs were held static. **(D)** Increase in MEP amplitude across all cycling conditions compared to legs static, No-tSCS.

To evoke consistent H-reflexes, a recruitment curve was first constructed to determine both the ascending and descending limb of the H-reflex amplitude (H_max_) curve, including the maximal H_max_. This was followed by finding the stimulation intensity that elicited a reflex that was approximately 70% of H_max_ on the ascending limb. In this range of intensity, M-wave co-occur with the H-reflex, which were needed as a guide for maintaining similar stimulation conditions across trials. The amplitude of maximal motor responses (M_max_) was recorded by averaging three supra-maximal stimulation trials where the amplitude of M-wave no longer increased, indicating that all motor axons are recruited (Pierrot-Deseilligny and Mazevet, 2000). M_max_ was used to normalize H-reflex values across all trials to allow for comparison between individuals. For the remainder of the experiment, the stimulation intensity was set to maintain a consistent small, but measurable M-wave amplitude (∼10% of M_max_) across trials to minimize antidromic effects (Figure 3B). The amplitude of the M-wave was monitored throughout the experiments and the stimulation amplitude was adjusted when necessary to ensure consistency in the evoked M-wave. Examples of FCR H-reflex responses (10 sweeps) from an individual participant are provided in Figures 4A–C and Figures 5A,B.

### Motor Evoked Potentials

To assess excitability of the corticospinal tract, TMS was applied to the contralateral motor cortex (single-pulse, monophasic) using a double cone coil to elicit motor evoked MEPs in the FCR muscle (Magstim2002, Magstim, Whitland, United States). To find the optimal coil position, stimulation was provided at multiple locations over the primary motor region of the forearm. The location that consistently produced the largest FCR MEP was then marked and maintained across all MEP trials. The same experimenter held the coil throughout the trials and care was taken to align the coil position with the marker. Each participant held a background contraction between 5 and 10% MVC, and an MEP recruitment curve was established by increasing the TMS intensity in increments of 5% maximal stimulator output, from a level where a minimal response was elicited to a level where the MEP amplitude reached its maximum and no longer increased in magnitude with increasing stimulation (MEP_max_). At each stimulus amplitude, two repetitions of the stimulus were delivered, and peak-to-peak amplitude as well as times of onset and offset were determined. The TMS intensity that generated ∼60% of MEP_max_ was chosen for comparison across experimental conditions so both facilitation and inhibition of corticospinal projections would be possible (Figure 3C). Examples of FCR MEP responses (10 sweeps) from an individual participant are provided in Figures 4D–F and Figures 5C,D.

A control assessment of MEP amplitude was repeated three times during the experimental protocol to ensure that cortical excitability or coil placement had not changed throughout the experimental session. These assessments ocurred before the first task, between the first and second task, and after all trials were completed to verify the ∼60% value MEP amplitude was maintained over time. Ten TMS pulses were delivered for each of the 8 experimental conditions.

### Electromyography

Muscle activity of four muscles in the left arm was recorded via electromyography (EMG) during each trial: FCR, extensor carpi radialis (ECR), biceps brachii (BB), and triceps brachii (TB). Muscle activity was recorded from surface Ag-AgCl electrodes placed on the muscle belly and recorded at a sampling rate of 2000 Hz using a CED 1401 analog to digital conversion board and Spike 2 associated software (Cambridge Electronic Design, Cambridge, United States). All EMG signals were amplified 1000x during data collection and band-pass filtered from 30 to 1000 Hz. The EMG signals were used to record H-reflexes and MEPs from the FCR muscle. EMG from the other three muscle groups was recorded to ensure that homonymous and heteronomous muscle activity remained constant and did not affect H-reflexes and MEPs of the FCR muscle.

### Data Analysis

The peak-to-peak amplitude of M-wave, H-reflex, and MEP, as well as baseline activity of the FCR muscle, were analyzed in a window of 400 ms (staring 100ms pre-stimulus, ending 300 ms post-stimulus) using an off-line custom-written MATLAB script (Matlab, Nantick, MA, United States). A window of 100ms pre-stimulus (−100 ms to 0ms relative to stimulus onset) was selected to calculate the baseline FCR and ECR EMG activity averaged over ten sweeps for each experimental condition. To obtain the value of pre-stimulus muscular contraction, the mean of the signal in this 100 ms window was calculated and subtracted from the whole trace to remove any offset in the signal. The pre-stimulus background activity was then rectified and calculated as the mean activity in the 100ms window. The peak-to-peak amplitude of post-stimulus H-reflex, M-wave, and MEP were calculated by averaging ten sweeps per condition (Figures 3B,C). The average values were then normalized to the value of M_max_ for H-reflex measurements and to the value of MEP_max_ for MEP measurements, obtained in a separate trial immediately before the initiation of the testing conditions. The post-stimulus window of analysis for each evoked response was selected based on visual inspection.

### Statistical Analysis

The amplitude of FCR H-reflexes, M-waves, and MEPs along with FCR/ECR pre-stimulus baseline activity were compared across different experimental conditions using repeated-measure ANOVA (rmANOVA). During the static task, the effects of condition (No tSCS, Cervical, Lumbar, and Combined) were compared for H-reflex, MEP, M-wave and baseline muscle activity with a 1 × 4 ANOVA. To determine directly the influence each tSCS condition had relative to when tSCS was not provided, FCR H-reflex and MEP data were compared as the percent change in amplitude relative to the No tSCS condition using a 1 × 3 ANOVA (% change Lumbar vs. Cervical vs. Combined). Similarly, during the cycling task, the effects of condition (Static No-tSCS, Cycle No-tSCS, Cycle Cervical, Cycle Lumbar, and Cycle Combined) were compared for H-reflex, MEP, M-wave, baseline muscle activity, and cycling cadence with a 1 × 5 ANOVA. The percent change in H-reflex and MEP modulation from Static No-tSCS to Cycle No-tSCS is used in the literature as a measure of interlimb connectivity (Zhou et al., 2018b). Therefore, to assess potential influences of tSCS on interlimb connectivity, FCR H-reflex and MEP data were compared as the percent change relative to no-tSCS using a 1 × 4 ANOVA (% change Cycle No-tSCS vs. Cycle Cervical vs. Cycle Lumbar vs. Cycle Combined). Significant effects were followed by pairwise comparisons corrected by Tukey’s HSD adjustment for multiple comparisons. Differences with *p* ≤ 0.05 were accepted as statistically significant. Descriptive statistics are shown as mean ± standard error, unless otherwise stated. All statistical analyses were performed with SPSS Statistics 20 (IBM, Chicago, IL, United States).

## Results

A two-way ANOVA showed a significant main effect for tSCS intensity across all conditions [*F*_(__3,__52__)_ = 3.428, *p* = 0.023]. *Post hoc* analysis indicated a significant difference in tSCS intensity between the two sites (*p* = 0.002), but no significant difference between intensities used during MEP and H-reflex assessment (*p* > 0.05). During the leg cycling task, participants aimed to maintain a 1 Hz (60 rpm) cadence. Across all trials, the average actual cycling cadence for participants during the H-reflex assessment was 59.25 ± 1.54 (mean ± SD) and 59.42 ± 1.04 during MEP assessment, and the cadence was not different across conditions (*p* > 0.05).

### Baseline Muscle Activity and Evoked Motor Responses Across Conditions

**FIGURE 6:**
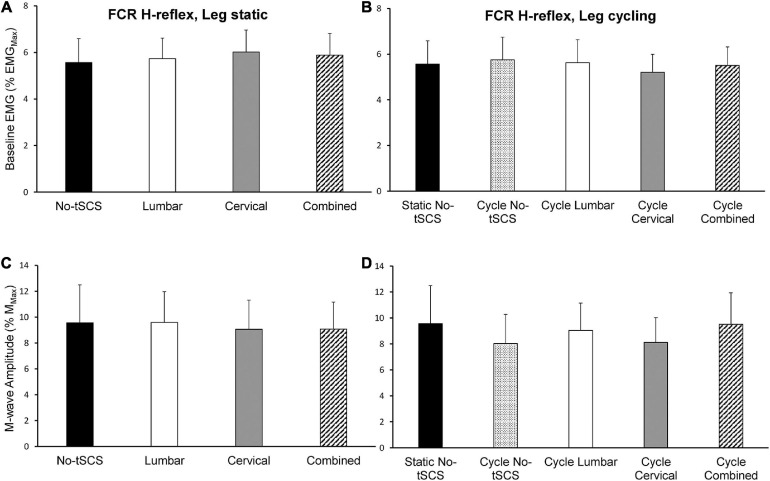
Baseline FCR muscle activity and M-wave amplitude during H-reflex assessments. The average baseline activity in the FCR muscle during H-reflex assessment (normalized to the maximal EMG activity during MVC) while the legs were **(A)** static and **(B)** cycling was similar indicating that a consistent contraction was held across all experimental conditions. The average peak-to-peak amplitude of the FCR M-wave was similar across all **(C)** static and **(D)** cycling conditions indicating that a similar direct motor response was evoked by the median nerve stimulus across all experimental conditions. Values are Mean ± SE.

The same level of background EMG was maintained across all conditions throughout the experiment. Moreover, baseline FCR and ECR muscle activity was maintained across tasks as there were no significant differences across all static and cycling conditions during both H-reflex and MEP assessments (Figures 6A,B, 8A,B). During the static task a 1 × 4 ANOVA indicated no difference in FCR [*F*_(__3,__42__)_ = 0.35, *p* = 0.786] or ECR [*F*_(__3,__42__)_ = 0.625, *p* = 0.603] baseline muscle activity between H-reflex conditions (Figure 6A). Across all static MEP assessment conditions, there was no difference in baseline FCR [*F*_(__3,__42__)_ = 0.807, *p* = 0.497] or ECR [*F*_(__3,__42__)_ = 0.589, *p* = 0.626] muscle activity (Figure 8A). During cycling trials, a 1 × 5 ANOVA revealed no difference in pre-stimulus FCR [*F*_(__4,__52__)_ = 0.508, *p* = 0.730] or ECR [*F*_(__4,__52__)_ = 0.833, *p* = 0.510] muscle activity during H-reflex assessment (Figure 6B). There was also no difference in FCR [*F*_(__4,__52__)_ = 0.124, *p* = 0.973] or ECR [*F*_(__4,__52__)_ = 0.321, *p* = 0.862] muscle activity during MEP cycling assessment (Figure 8B).

During H-reflex assessment, there was no difference in FCR M-wave amplitude between conditions [static: *F*_(__3,__42__)_ = 0.112, *P* = 0.953] (Figure 6C). Also, no significant difference was found in M-wave amplitude during H-reflex cycling assessment across all conditions [*F*_(__4,__52__)_ = 0.640, *p* = 0.637] (Figure 6D). Thus, the descending input to the motor pool and effects of reciprocal inhibition from the antagonist muscle group were similar across all tasks and conditions. Moreover, a similar direct motor response during FCR H-reflex assessments was maintained irrespective of task and condition.

### Effect of tSCS on H-Reflex Excitability

**FIGURE 7:**
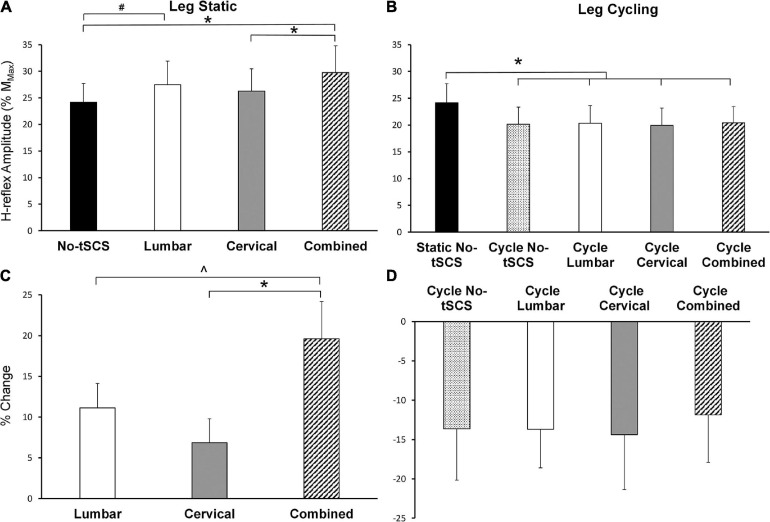
Effect of tSCS on FCR H-reflex amplitude while the legs were static or cycling. **(A)** Average H-reflex peak-to-peak amplitude during the application of tSCS while the legs were held static. Combined tSCS increased the amplitude of the H-reflex significantly more than No-tSCS and Cervical tSCS. Lumbar tSCS significantly increased the amplitude of the H-reflex compared to No-tSCS. **(B)** Average peak-to-peak H-reflex amplitude during the application of tSCS while the legs were cycling. There was a significant reduction in H-reflex amplitude for all cycling conditions regardless of tSCS site compared to the legs static, No-tSCS condition. **(C)** Percent increase in H-reflex amplitude during Lumbar, Cervical, and Combined tSCS while the legs were static relative to No-tSCS. The% increase in H-reflex amplitude with Combined tSCS was significantly larger than that with Lumbar or Cervical tSCS. **(D)** Percent decline in H-reflex amplitude during all tSCS conditions while the legs were cycling relative to the legs static, No-tSCS condition. *p < 0.05, ^#^*p* = 0.059, ^*p* = 0.053. Values are Mean ± SE.

During the static tasks, a 1 × 4 rmANOVA indicated a significant main effect of condition on H_max_ [*F*_(__3,__42__)_ = 6.79, *p* < 0.001]. *Post hoc* pairwise comparisons revealed a significant facilitation of FCR H-reflex during Combined tSCS (29.8% M_max_; *p* < 0.001; *d* = 0.34) compared to No-tSCS (24.2% Mmax) (Figure 7A). H_max_ during Combined tSCS was also significantly greater than Cervical (26.2% M_max_; *p* = 0.041) but not different than Lumbar tSCS (*p* > 0.05) (Figure 7A). Lumbar tSCS approached statistical significance (29.1% M_max_; *p* = 0.0596; *d* = 0.22) compared to No-tSCS (24.8% M_max_). Cervical and lumbar tSCS was not significantly different from No-tSCS (*p* > 0.05). A 1 × 3 rmANOVA indicated a significant main effect of condition on% change in H_max_ [*F*_(__2,__28__)_ = 7.09, *p* = 0.004]. The percent increase in H_max_ relative to No-tSCS was significantly larger for Combined tSCS (19.6% increase) than for Cervical (6.9% increase; *p* = 0.003; *d* = 0.89) and approached significance compared to Lumbar tSCS (11.1% increase; *p* = 0.053; *d* = 0.59) (Figure 7C).

During the cycling task, a 1 × 5 rmANOVA indicated a significant main effect of condition on H_max_ [*F*_(__4,__52__)_ = 3.80, *p* = 0.009]. All cycling conditions significantly suppressed the amplitude of the H-reflex compared to the legs static No-tSCS condition, including cycling with No-tSCS (13.6% decrease; *p* = 0.024; *d* = 0.32), cycling with Lumbar (13.6% decrease; *p* = 0.035; *d* = 030.), cycling with Cervical (14.3% decrease; *p* = 0.015; *d* = 0.33), and cycling with Combined (11.8% decrease, *p* = 0.042; *d* = 0.30) tSCS (Figure 7B). However, there was no difference in the percent reduction in H_max_ between any of the cycling conditions relative to the legs static No-tSCS condition (*p* > 0.05; Figure 7D) indicating that tSCS likely did not influence interlimb connectivity.

### Effect of tSCS on MEP Excitability

**FIGURE 8:**
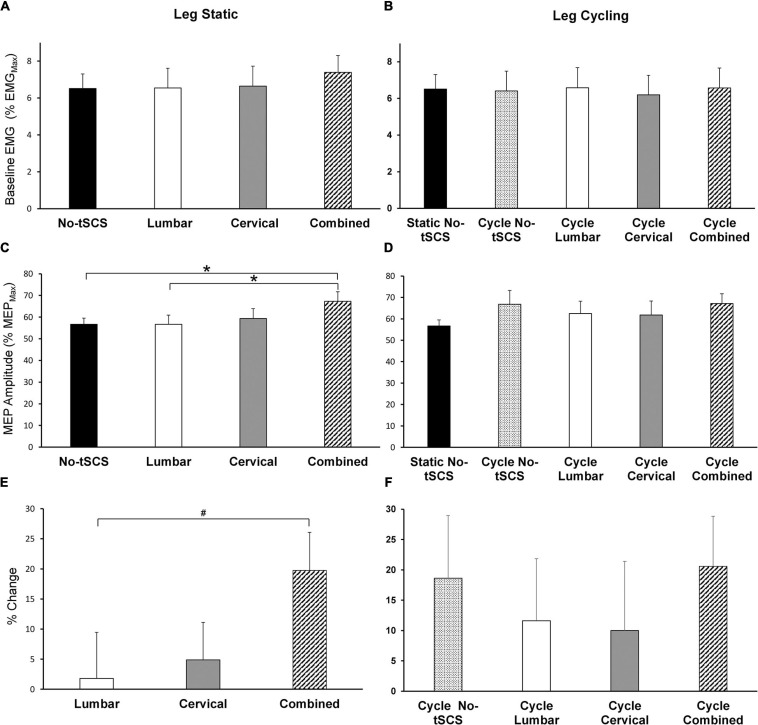
Effect of tSCS on FCR MEP amplitude while the legs were static or cycling. Average amplitude of baseline activity during MEP assessments while the legs were **(A)** static and **(B)** cycling was similar across all conditions. **(C)** Average MEP peak-to-peak amplitude during tSCS while the legs were held static. Combined tSCS significantly increased the amplitude of the MEPs relative to No-tSCS and Lumbar tSCS. **(D)** Average MEP amplitude during tSCS while the legs were cycling. There was no significant difference in the amplitude of MEPs compared to the static, No-tSCS condition for all cycling conditions. **(E)** Percent increase in MEP amplitude during Lumbar, Cervical and Combined tSCS relative to the legs static, No-tSCS condition. The% increase in MEP amplitude with Combined tSCS was significantly larger than that with Lumbar tSCS. **(F)** Percent increase in MEP amplitude during leg cycling relative to the legs static, No-tSCS condition. *p < 0.05, ^#^*p* = 0.056. Values are Mean ± SE.

Average baseline EMG activity in FCR remained constant across all leg static (Figure 8A) and leg cycling (Figure 8B) conditions. As well, static No-tSCS MEP amplitude did not change from the beginning to the middle to the end of the experiment. Together this demonstrates that the background corticospinal drive was consistent throughout the experiment and corticospinal excitability was similar when assessed at multiple timepoints under the same conditions. During the legs static task, there was a significant main effect of condition on MEP amplitude revealed by a 1 × 4 ANOVA [*F*_(__3,__42__)_ = 3.28, *p* = 0.031]. *Post hoc* pairwise comparisons showed that Combined tSCS significantly facilitated MEP amplitude relative to Lumbar (*p* = 0.047; *d* = 0.66) and No-tSCS (*p* = 0.047; *d* = 0.76) (Figure 8C). Lumbar and Cervical tSCS alone did not significantly alter the amplitude of the MEP relative to No-tSCS (*p* > 0.05) and Combined tSCS was not significantly different than Cervical tSCS (*p* > 0.05). There was a significant main effect of the percent increase in MEP amplitude from No-tSCS [*F*_(__2,__26__)_ = 3.39, *p* = 0.049] (Figure 8E). Combined tSCS (19.8% increase) facilitated an increase in MEP amplitude that approached significance compared to Lumbar tSCS (1.8% increase *p* = 0.056; *d* = 0.68). Combined was not different than Cervical tSCS (4.9% increase; *p* > 0.05; *d* = 0.63) and there was no difference between Lumbar and Cervical tSCS (*p* > 0.05).

Figures 8D,F summarize the changes in corticospinal excitability while the legs were cycling. All cycling conditions increased the amplitude of MEPs relative to the static, No-tSCS condition; however, there were no significant differences in MEP amplitude between conditions [*F*_(__4,__52__)_ = 3.80, 1.579, *p* = 0.194] (Figure 8D). Relative to the legs static, No-tSCS condition, corticospinal excitability was facilitated during cycling without tSCS (18.6% increase), as well as during cycling with Lumbar (11.6% increase), Cervical (10.0% increase), and Combined tSCS (20.6% increase) (Figure 8F).

## Discussion

### Overview

Although tSCS provides functional improvements in the upper and lower limbs in people with a SCI, there is a continued lack of knowledge regarding the neuromodulation in sensorimotor circuitry that occurs with its use. The present results demonstrate that tSCS can alter spinal reflex and corticospinal excitability in neurologically intact individuals, observed as changes in the amplitude of the FCR H-reflex and MEP. In our previous work, cervical tSCS significantly suppressed the activity of lumbar networks in a manner similar to the effect produced by rhythmic arm cycling. Thus, we hypothesized that lumbar tSCS would suppress the FCR H-reflex as well, signifying bidirectional tSCS effects on the cervico-lumbar networks. We also expected that with simultaneous cervical and lumbar tSCS, these neuromodulatory effects on the H-reflexes may be canceled out. Moreover, based on the findings in Benavides et al. (Benavides et al., 2020), we expected that cervical tSCS would suppress the corticospinal drive to the FCR muscle, and that combined cervical and lumbar tSCS would produce an even larger suppression.

Contrary to our hypothesis, during the legs static task, lumbar tSCS facilitated the FCR H_max_ by 11.1% (relative to No tSCS), while cervical tSCS altered the FCR H_max_ by 6.8%. Interestingly, combined cervical and lumbar tSCS significantly enhanced the facilitation of the FCR H-reflex (by 19.6%) compared to either site alone. Moreover, while neither cervical nor lumbar tSCS altered MEP amplitude alone (+4.9% and 1.8% relative to static tSCS), combined tSCS significantly increased MEP amplitude by 19.7% compared to No tSCS.

Leg cycling alone significantly suppressed the FCR H-reflex relative to legs static, No-tSCS (by 13.6%) while facilitating MEP amplitude by 18.6%. tSCS was unable to further alter H-reflex or MEP excitability in any condition. This indicates that in neurologically intact individuals where interlimb coordination and corticospinal tract are intact, the effect of leg cycling on cervico-lumbar coupling and corticospinal drive was unable to be impacted significantly with the intensity of tSCS used. This study demonstrates, for the first time, that tonic activation of spinal cord networks through multiple sites of tSCS provides a facilitation of both spinal reflex and corticospinal pathways.

During all trials, participants maintained a consistent baseline muscle contraction in the FCR muscle across all tasks and conditions (Figures 6A,B, 8A,B) to ensure that changes in voluntary contraction did not influence the amplitude of H-reflex (Matthews, 1986). Moreover, the amplitude of the direct M-wave, which is a neural signature of the amount of recruited efferent axons (Dietz et al., 1990; Brooke et al., 1997), was carefully maintained across tasks and conditions for during H-reflex assessments (Figures 6C,D). Disynaptic reciprocal inhibition can also influence the amplitude of H-reflex (Petersen et al., 1999; Morita et al., 2001; Zehr, 2002), making it important that there were no significant differences in ECR baseline muscle activity occurred across conditions or tasks in the current investigation Furthermore, background FCR muscle activity remained unchanged across all MEP measurements (Figure 8), which shows that the motorneuron excitability was held constant. Thus, it is unlikely that the level of descending drive, the number of recruited axons, or reciprocal inhibition underlie the modulation seen in this study in H-reflexes and MEPs in the forearm with tSCS.

### tSCS Alters Excitability of Remote Segments of the Spinal Cord

Results from the current investigation highlight that tSCS can alter excitability across multiple segments of the spinal cord. Importantly, multi-site (i.e., Combined) tSCS led to a 19.6% increase in H_max_, while Lumbar tSCS increased the amplitude of FCR H-reflex relative to No-tSCS by 11.1% with the legs static (Figure 7C).

It may be possible that non-invasive spinal stimulation activates the spinal motor pools by increasing sensory inputs through Ia afferents (Sayenko et al., 2019). Our previous investigation determined that tonic activation of the cervical region through tSCS suppresses the amplitude of the soleus H-reflex (Barss et al., 2019) to a similar extent as that produced by rhythmic arm cycling (Zhou et al., 2018b), indicating that tSCS may also engage propriospinal interneuronal connections exerting effects on multiple segments of the spinal cord. Therefore, it was tempting to assume this suppressive effect would be bidirectional: tonic activation of the lumbar networks of the spinal cord by tSCS would reduce the amplitude of the H-reflex in FCR similarly to the suppressive effect rhythmic leg cycling has on the FCR H-reflex. While the present findings indicate that tSCS alters excitability across multiple segments of the spinal cord, the resulting facilitation in H_max_ with lumbar tSCS and suppression with leg cycling suggest that separate networks are responsible for the effects. The current investigation is unable to identify specific pathways or sites responsible for the disinhibition of the FCR H-reflex. However, facilitation of the H-reflex pathway through tSCS may be due to reduced Ia presynaptic inhibition or facilitation of the motor pool through activation of posterior root afferents and interneuronal projections (Hofstoetter et al., 2018). With the current methodology, it also remains possible that the stimulation of skin itself may alter cutaneous afferent transmission, altering the excitability of the spinal cord. The potential role of the skin with tSCS remains an important avenue to explore in future work (Beekhuizen and Field-Fote, 2005).

### tSCS Does Not Alter H-Reflex Amplitude During Rhythmic Leg Cycling

It is well established that rhythmic movements of the arm or leg modulate spinal reflex excitability of the remote limb (Palomino et al., 2011), and these reciprocal neural connections are damaged after SCI (Zhou et al., 2018b) and stroke (Klarner et al., 2016b). However, spared neural connections are viable to be retrained by a rhythmic A&L cycling intervention to restore intersegmental linkages (Klarner et al., 2016b). Previously, our group assessed the benefits of actively engaging the arms rhythmically with the legs in a rehabilitation paradigm. Twelve weeks of simultaneous A&L cycling improved cervico-lumbar coupling, exemplified by the restoration of a significant reduction in the soleus H_max_ during dynamic arm cycling in study volunteers with incomplete SCI (Zhou et al., 2018b).

Evidence suggests that modulation of H_max_ in the FCR muscle during rhythmic leg cycling (Nakajima et al., 2013) and in the soleus muscle during rhythmic arm cycling (Frigon et al., 2004) likely arise from elevated presynaptic inhibition of Ia afferent terminals. Thus, locomotor circuits of the cervical and lumbar spinal cord responsible for generating rhythmic movements act on presynaptic interneurons, which at least in part, reduces the transmission from Ia afferents onto spinal motoneurons. Interlimb coupling is composed of long descending and ascending propriospinal interneurons (Dietz, 2002; Frigon, 2017) mediating coordination of the locomotor control centers of the upper and lower extremities, and play a role in gating the excitability of reflex pathways (Dietz, 2002; Huang and Ferris, 2009). The lack of soleus H-reflex suppression during arm cycling after stroke and incomplete SCI is attributed to disruption or abolition of propriospinal networks (Barzi and Zehr, 2008; Zhou et al., 2018b). While A&L cycling training has been shown to reengage these connections, it remains unknown whether tSCS can actively engage previously inaccessible networks to potentially incite further Hebbian plasticity and improve outcomes.

As expected, we found that leg cycling significantly suppresses the amplitude of the H-reflex relative to the leg static, No tSCS condition (Figure 7B), a finding verified by various investigations (Nakajima et al., 2013; Zehr et al., 2016; Zhou et al., 2018b). Strengthened presynaptic inhibition projecting to Ia cervical afferent terminals is likely the primary contributor to this suppression, although reciprocal and recurrent inhibition may also contribute to the effect (Shefner et al., 1992; Nielsen et al., 1995; Petersen et al., 1999). Here, cycling trials with tSCS (Cervical, Lumbar, and Combined) did not alter excitability beyond what was produced by cycling alone (Figure 7B). The percent decline in H_max_ relative to the legs static, No-tSCS condition was similar among all cycling trials, suggesting the suppressive effect of cycling in a neurologically intact population may be too strong; thus, overriding the impact caused by tonic activation of the spinal cord for all cycling conditions with tSCS. It remains vital for future investigations to determine if tSCS can influence interlimb coupling after neural injury when cervico-lumbar connectivity is impaired.

### Combined tSCS Provides a Non-linear Facilitation of MEP Amplitude

A crucial finding of this project was the effect of tSCS on the excitability of the corticospinal tract as tested by MEPs produced with TMS. Simultaneous tSCS at the lumbar and cervical sites (Combined) significantly increased corticospinal transmission to the FCR muscle compared to static No-tSCS and Lumbar tSCS conditions (Figure 8C). This provides novel evidence that multiple sites of tSCS converge to facilitate corticospinal transmission (19.7%) to a greater extent than lumbar and cervical tSCS alone (6.7%). This increase in the amplitude of MEPs could be due to reinforced projection of corticospinal axons onto spinal motoneurons (Rothwell et al., 1994). Therefore, proprioceptive inputs generated by tSCS delivered to spinal motor neurons may be the main contributor to the facilitation of MEPs to the FCR muscle. Recently, a study determined that single site tSCS applied with a 5kHz carrier frequency at the C5-C6 level facilitated the amplitude of CMEPs, but did not increase the amplitude of the MEPs (Benavides et al., 2020). This was accompanied by an increase in the level of short-interval cortical inhibition (SICI). While this suggests that alterations in spinal circuitry are likely the target of tSCS for facilitating corticospinal excitability, it is important to note that those results occurred after 20 min of tSCS and were assessed over a long duration compared to the results of the current investigation.

### tSCS Does Not Alter Corticospinal Excitability During Rhythmic Leg Cycling

Across all cycling trials, with or without tSCS, there was a general facilitation of MEPs relative to the static condition, but with no significant differences between conditions. Both cortical and spinal mechanisms are involved in modulating corticospinal excitability during rhythmic movements of the legs (Zhou et al., 2017). Propriospinal neurons that link the cervical and lumbar locomotor networks, transmit locomotor commands from supraspinal locomotor regions (Cowley et al., 2008; Laliberte et al., 2019); thus, corticospinal commands propagating along these propriospinal connections can possibly be modulated, and may have partially played a role in the facilitation of FCR MEPs. Additionally, owing to the overlap of the representations of the arm and leg muscle representations in the pre-motor and supplementary motor areas of the human cortex that project to primary motor cortex, modulation of forearm corticospinal excitability occurs during cyclical ankle movements enhancing hand-foot coordination (Byblow et al., 2007). Furthermore, voluntary rhythmic activity of the foot causes fluctuations in the activity of cortical regions projecting to the forearm muscles along with full activation of the foot-associated cortical area (Baldissera et al., 2002). Hence, intracortical connections and changes in intracortical excitability may contribute to the increased FCR MEP amplitude during leg cycling.

### Mechanisms Involved in Transcutaneous Spinal Cord Stimulation

Neuromodulation of spinal circuitry through the use of tSCS improved functions such as increased pinch and hand grip force, strength and dexterity of the upper extremity, stepping, standing, posture, mitigation of spasticity below the level of injury, and regulation of blood pressure (Gerasimenko et al., 2015a,b; Balykin et al., 2017; Gad et al., 2018; Inanici et al., 2018; Sayenko et al., 2019). While tSCS can facilitate motor retraining, the neural mechanisms and pathways responsible for the effect have yet to be comprehensively identified. The principal mechanism by which tSCS non-invasively activates inaccessible neuronal networks of the spinal cord likely includes recruitment of afferent fibers (large-to-medium) of the posterior root (Sayenko et al., 2019). It has also been suggested that tSCS may share similar physiological principles to eSCS, although tSCS targets a broader network within the spinal cord (Gad et al., 2018). In a comparative study, EMG characteristics of evoked responses elicited by tSCS and eSCS in multiple leg muscles including rectus femoris, biceps femoris, and tibialis anterior were analyzed. Both tSCS and eSCS produced reflex-based responses as manifested in post-activation depression of responses. This shared nature was ascribed to similarities in latency, peak-to-peak amplitudes, and waveform of the evoked EMG responses (Hofstoetter et al., 2018). In addition, computer simulation of the posterior root fibers demonstrated that tSCS initiated action potentials in those fibers at their entry into the spinal cord or at their exit from the spinal canal, replicating the effect of eSCS (Ladenbauer et al., 2010). Although the activation of posterior root afferents has been identified as a contributing mechanism of tSCS, other pathways are likely involved such as activation of interneuronal circuits via synaptic projections (Minassian et al., 2007) or enhances in the efficacy of cortico-motoneuronal synapses (Inanici et al., 2018).

An important feature of the present study is the implementation of 10 kHz burst carrier frequency. Previous investigations have highlighted that for distal muscles, the occurrence probability of antidromic collisions is very high for afferent firing rates of 30 impulses per second (Imp/s) at 30Hz tSCS (Formento et al., 2018). This could lead to a lack of proprioceptive information being available during locomotor training if incorporated and could limit the clinical relevance. However, including burst stimulation is thought to mitigate the cancelation of proprioceptive information enabling greater control of motoneuron activity (Schu et al., 2014) and has become an important component in many applications of tSCS (Inanici et al., 2018; Benavides et al., 2020; Pradarelli et al., 2020). Our observations help to elucidate contributing mechanisms involved in the use of tSCS which may facilitate its targeted use to reengage previously inaccessible circuitry to improve motor function after neurological injury.

### Limitations and Future Directions

Prior to the widespread clinical use of tSCS, vital steps remain to be addressed including a detailed understanding of the circuitry being recruited and its influence on excitability both the neurologically intact and impaired nervous system (e.g., SCI, stroke, MS, etc.). It remains critical to evaluate whether tSCS can alter cervico-lumbar connectivity during cycling in clinical populations in which these connections are impaired. Importantly, our stimulation intensity in a neurologically intact population is much less than generally used in a clinical setting. Our average stimulation intensity for cervical and lumbar tSCS were 51 mA and 43 mA, whereas recent investigations after SCI have used stimulation intensities in the range of 80–120 mA for the same sites of stimulation. Thus, it will be vital for future experiments to determine if higher amplitudes of stimulation delivered to a spinal cord with impaired cervico-lumbar connectivity provides larger H-reflex and corticospinal effects compared to those in the current investigation. Moreover, the current investigation chose tSCS intensities based on the participants’ subjective maximal tolerable intensity without pain. It is common practice for many other groups to base their tSCS intensity on evoked motor thresholds which makes comparison between studies possible. Nonetheless, while the lack of evoked potentials limits direct comparison to other studies, the comparison between conditions in the current study remains valid. Combining tSCS with A&L cycling for people with SCI may facilitate improved motor outcomes such as walking, standing and balance. Further exploration into how best to incorporate tSCS into rehabilitation, while carefully considering other neuromodulation techniques including invasive stimulation, implantable technologies, exoskeletons, and assistive devices (bionic gloves, functional electrical stimulation, walkers, etc.) will be necessary considerations as the field continues to move forward at an increasingly rapid pace.

## Data Availability Statement

Processed data from this work can be accessed at http://dx.doi.org/10.34945/F5B59T. Unprocessed data are available upon request from the corresponding author.

## Ethics Statement

The studies involving human participants were reviewed and approved by the Human Research Ethics Board, University of Alberta. The participants provided their written informed consent to participate in this study.

## Author Contributions

BP, TB, and VM contributed to the inception of the study and experimental design, contributed to the interpretation of the results, and provided revisions. VM approved the final draft of the manuscript. BP and TB collected and analyzed the data and drafted the early versions of the manuscript. All authors contributed to the article and approved the submitted version.

## Conflict of Interest

The authors declare that the research was conducted in the absence of any commercial or financial relationships that could be construed as a potential conflict of interest.
